# Aggression mediates the association between problematic smartphone use and cyberbullying perpetration: a 6-year longitudinal study of Korean adolescents

**DOI:** 10.3389/fpubh.2026.1820848

**Published:** 2026-06-09

**Authors:** Hyunjoo Na, Hye Seung Choi, Il Hyun Lee, Gyungjoo Lee

**Affiliations:** 1 College of Nursing, The Catholic University of Korea, Seoul, Republic of Korea; 2 Department of Nutrition and Food Science, University of Maryland, College Park, Maryland, MD, United States; 3 StatEdu, Institute of Statistics, Iksan, Republic of Korea

**Keywords:** adolescent behavior, aggression, cyberbullying perpetration, parenting, problematic smartphone use

## Abstract

**Introduction:**

Cyberbullying perpetration is a form of interpersonal violence among adolescents that is associated with mental health risk factors and outcomes, yet the longitudinal mechanisms linking problematic smartphone use (PSU) to cyberbullying remain unclear. Guided by the General Aggression Model, this study examined whether aggression mediates the relationship between PSU and cyberbullying perpetration and whether parental structuring moderates this indirect pathway.

**Methods:**

The study utilized six waves of nationally representative panel data from 2,242 Korean adolescents participating in the Korean Children and Youth Panel Survey. Generalized estimating equation models were applied to test longitudinal mediation and moderated mediation across adolescence. PSU was specified as the independent variable, aggression as the mediator, cyberbullying perpetration as outcome, and parental structuring as the moderator. Covariates included sleep duration, leisure time, physical activity, smartphone usage time, and peer relationships.

**Results:**

PSU was significantly associated with increased aggression over time, which subsequently was associated with increased cyberbullying perpetration, indicating an indirect pathway. Parental structuring was associated with a stronger PSU -aggression relationship during adolescence rather than buffering it. Aggression functioned as a key mechanism linking PSU to cyberbullying perpetration, and this pathway was stronger among adolescents reporting higher levels of parental structuring.

**Discussion:**

These findings suggest that parental regulation of adolescent smartphone use alone may be insufficient, and that prevention of cyberbullying requires multilevel interventions integrating school- and community-based programs and developmentally appropriate strategies targeting online risk behaviors.

## 1 Introduction

Cyberbullying perpetration, defined as the deliberate infliction of verbal or psychological harm on peers through digital means, is a critical public health concern among adolescents worldwide (1). A meta-analysis of globally conducted studies published between 2019 and 2022 reported a pooled prevalence of 15.5% for cyberbullying victimization among children and adolescents (2). In South Korea, nearly one in five adolescents (19.3%) reported perpetrating cyberbullying in 2023 (3). Features of online environments, such as perceived anonymity and reduced physical constraints, may facilitate aggressive behaviors by lowering social inhibition, enabling cyberbullying to extend across temporal and spatial boundaries and reach broad audiences rapidly (4, 5). Adolescent cyberbullying perpetrators often have prior involvement in traditional bullying as either perpetrators or victims (6, 7), and their behaviors are associated with significant psychological consequences, including social anxiety, diminished self-esteem, and suicidal ideation (8). Therefore, understanding the mechanisms that drive cyberbullying perpetration is essential for protecting adolescents' mental health.

Cyberbullying is closely associated with smartphone use, which facilitates easy access to online environments. As smartphones have become indispensable tools for acquiring information and forming social connections, offering adolescents opportunities for learning, communication, and skill development as they are integrated into daily routines. However, alongside these benefits, problematic smartphone use (PSU) has emerged as a closely related behavioral concern among adolescents. PSU refers to a pattern of excessive or maladaptive smartphone use characterized by daily-life disturbance, withdrawal, tolerance, and a preference for virtual over real-life interactions (9). According to recent Korean national data, the overall prevalence of PSU decreased from 23.6% in 2022 to 22.9% in 2024, indicating a continuous decline over the past three years. However, unlike other age groups, Korean adolescents aged 10–18 years showed an opposite trend, with prevalence increasing from 40.1% to 42.6% over the same period (10). PSU has also been recognized as a growing concern in other countries, with evidence indicating increasing levels of problematic internet use across diverse cultural contexts (11). In addition, PSU is associated with a range of psychosocial and behavioral difficulties, including increased aggression, emotional distress, and risk behaviors among adolescents (12, 13). As PSU has been linked to aggressive behaviors such as cyberbullying, the association between PSU and cyberbullying perpetration raises critical questions about the underlying mechanisms.

Recent empirical evidence suggests that PSU is positively associated with both aggression and cyberbullying perpetration among middle and high school students (14, 15). The General Aggression Model provides a theoretical framework for understanding these associations (16). This model posits that aggression develops through the interaction of situational variables and individual characteristics, shaping an individual's internal state and facilitating or inhibiting aggressive behavior. Within this framework, PSU functions as a situational risk factor by increasing exposure to hostile online cues and opportunities for digital aggression, thereby fostering aggressive knowledge structures (17). Moreover, interpersonal and behavioral vulnerabilities such as poor peer relationships and insufficient sleep have been linked to heightened PSU and aggression, intensifying the risk of cyberbullying perpetration in early adolescents (18). Despite growing evidence linking PSU to cyberbullying, limited longitudinal research has examined the mechanisms underlying this association, particularly the mediating role of aggression. Consistent with the General Aggression Model, examining this pathway is important for understanding how PSU may contribute to cyberbullying perpetration during adolescence.

Parenting also plays a pivotal role in adolescents' media-related problem behaviors, including PSU and cyberbullying (17, 19). Parental structuring is defined as guidance that helps children attain positive outcomes and avoid negative consequences across social and physical contexts; such guidance encompasses such practices as explaining, coaching, and educating to facilitate problem-solving and planning (20). This approach, a form of inductive discipline, is widely recognized as an effective strategy for promoting moral development (21). Within the context of cyberbullying, parental structuring may moderate the relationships among PSU, aggression, and cyberbullying perpetration. Parent-focused interventions have been effective in reducing cybervictimization, although their long-term effects on cyberbullying perpetration appear to be limited (22). Although parental structuring is known to guide and constrain children's online behaviors, its moderating role in the relationship between PSU, aggression, and cyberbullying perpetration during adolescence remains unclear. Existing studies have primarily focused on the protective role of parental monitoring or control, while limited longitudinal research has examined how parental structuring functions in the pathway linking PSU to cyberbullying perpetration through aggression. Therefore, longitudinal examination of the moderating role of parental structuring in this pathway is warranted.

Adolescents' online behaviors are shaped not only by psychological factors but also by daily lifestyle and interpersonal relationships. Sleep duration, leisure and physical activity time, smartphone usage time, and peer relationships have all been associated with aggression and cyberbullying involvement (18, 23, 24). Consequently, these variables should be controlled when examining pathways linking PSU, aggression, and cyberbullying perpetration. The current study addressed existing research gaps by investigating the longitudinal relationships among PSU, aggression, and cyberbullying perpetration. To do so, we used six waves of nationally representative panel data from the Korean Children and Youth Panel Survey (KCYPS) that spanned the critical developmental transition from middle to high school. Guided by the General Aggression Model, this study examined whether higher PSU would be positively associated with cyberbullying perpetration through increased aggression and whether parental structuring would moderate the association between PSU and aggression across six years of adolescent development. In both analyses, we controlled for potentially relevant lifestyle and peer-related behavior variables.

## 2 Methods

### 2.1 Study design and data

We conducted a secondary analysis of KCYPS data collected annually from 2018 to 2023 (25). Using a multiple-point, prospective panel design, South Korea's National Youth Policy Institute conducted the KCYPS to generate a nationally representative set of panel data that comprehensively captures changes in the growth and development of children and adolescents. The 2018 KCYPS sample consisted of students enrolled in their first year of middle school. A total of 2,590 first-year middle school students in 333 schools were selected using a multistage stratified cluster sampling technique to ensure regional representation. The sampling framework included three levels: provinces and metropolitan areas as primary units, schools selected via probability-proportional-to-size sampling as secondary units, and specific classes as tertiary units. Some 17 provinces and cities were stratified, and schools were randomly selected within each region based on proportionate probability sampling. The sample was proportionally distributed across these regions to reflect the population distribution, supporting generalizability of results to the entire population of South Korean children and adolescents. Over the 6-year study period, a self-report survey and face-to-face interviews were conducted each year.

The first wave of data was collected from South Korean students in their first year of middle school (8th grade) in 2018. These students, aged 12 to 14, responded to the survey, which included questions on cyberbullying. They continued to complete assessments annually until they reached their third year of high school (12th grade) in 2023. Of the 2,590 participants (1,405 boys and 1,185 girls), only those who provided responses to the cyberbullying perpetration measure in at least three of the six annual waves; 2,242 individuals met this criterion. This inclusion criterion was applied to ensure sufficient repeated observations within individuals and to capture meaningful longitudinal patterns for longitudinal analysis. Among the participants at baseline, 27 (1.2%) were 12 years old, 2,206 (98.4%) were 13 years old, and 9 (0.4%) were 14 years old.

The dataset was obtained from the National Youth Policy Institute and, as a publicly available de-identified secondary dataset, was deemed exempt from Institutional Review Board (IRB) review and approved accordingly prior to analysis.

### 2.2 Study variables

#### 2.2.1 Main variables

*Problematic Smartphone Use (PSU)* was examined using the Smartphone Addiction Proneness Scale developed by Kim et al. (9). The scale includes 15 items assessing four dimensions of problematic smartphone use: daily-life disturbance, virtual life orientation, withdrawal, and tolerance. Responses were rated on a four-point Likert scale (1 = strongly disagree; 4 = strongly agree). Higher scores indicated higher levels of dependence on smartphones. In this study, the scale's Cronbach's alpha ranged from 0.76 to 0.83 across waves.

*Aggression* was assessed using the aggressive behavior subdomain of the Emotional or Behavioral Problems Scale (EBPS), which consists of five subdomains: difficulty in making friends, attention deficit hyperactivity, somatic symptoms, undesirable expression of emotion, and aggressive behavior (26). In the present study, six modified items from the aggressive behavior subdomain, as adapted for the KCYPS survey, were used. Aggression reflected broader emotional and behavioral aggression tendencies, including irritability, anger, and reactive aggressive responses. Responses were rated on a four-point Likert scale (1 = strongly agree; 4 = strongly disagree), reverse-coded, and summed. Higher scores indicated higher levels of aggression. In this study, the scale's Cronbach's alpha ranged from 0.83 to 0.86 across waves.

*Parental structuring* was evaluated using the Korean version of the Parents as Social Context Questionnaire for Adolescents developed by Kim and Lee (27). The scale contains 24 items in sub-dimensions, including warmth, autonomy, support, structuring, rejection, coercion, and chaos. Data for four items within the parental structuring dimension were used in this study. Parental structuring refers to the act of providing children with necessary, consistent guidance and rules to navigate their lives. Responses were rated on a four-point Likert scale (1 = strongly agree; 4 = strongly disagree). Higher scores reflected a higher level of structured guidance provided by parents. In this study, the items' Cronbach's alpha ranged from 0.73 to 0.81 across waves.

*Cyberbullying perpetration* was evaluated using the Cyberbullying Profile Scale developed by Lee et al. (28). This scale has 15 questions reflecting various types of cyberbullying perpetration. Respondents were asked to report their frequency of engagement in each type over the previous 12 months. Responses were rated on a six-point Likert scale (0 = never; 5 = many times weekly). Higher scores indicated greater frequency of cyberbullying perpetration. In this study, the scale's Cronbach's alpha ranged from 0.51 to 0.93 across waves.

#### 2.2.2 Control variables

*Demographic characteristics* included sex, residential area, perceived socioeconomic status (SES), and primary caregiver. These control variables were measured at baseline.

*Average sleep duration per day* was calculated using self-reported bedtimes and wake-up times on both weekdays and weekends. The average sleep duration per day was then utilized as a control variable.

*Leisure and physical activity time (weekday/weekend)* was assessed using a seven-point ordinal scale: (1) not at all, (2) less than 30 min, (3) 30 min to less than 1 h, (4) 1 h to less than 2 h, (5) 2 to less than 3 h, (6) 3 to less than 4 h, and (7) 4 h or more. Higher scores indicated greater leisure and physical activity time.

*Smartphone usage time (weekday/weekend)* was assessed using a seven-point ordinal scale: (1) not at all, (2) less than 30 min, (3) 30 min to less than 1 h, (4) 1 h to less than 2 h, (5) 2 to less than 3 h, (6) 3 to less than 4 h, and (7) 4 h or more. Higher scores indicated greater smartphone usage time.

*Peer relationships* were assessed using the Peer Relationship Quality Scale for Adolescents developed by Bae et al. (29). The scale consists of 13 items, and responses were rated on a four-point Likert scale (1 = strongly disagree; 4 = strongly agree). Example items include “I spend time with friends,” “I am comfortable talking to my friends about myself,” “I often experience conflicts of opinion with my friends,” and “My friends show little interest in my problems or hardships.” Negative items were reverse-coded, and item scores were summed. Higher scores indicated better peer relationships. In this study, the scale's Cronbach's alpha ranged from 0.65 to 0.74 across waves.

*Peer aggression behaviors* were assessed using selected self-report items from a delinquent behavior scale developed by Kim et al. (30), reflecting harmful interpersonal behaviors directed toward peers. The items included severe teasing, group exclusion (ostracism), physical aggression, threatening others, stealing money or belongings, sexual harassment, and verbal abuse. Responses were rated on a six-point Likert scale (0 = never; 5 = many times weekly). Higher scores indicated greater frequency of peer aggression behaviors. In this study, the scale's Cronbach's alpha ranged from 0.56 to 0.95 across waves.

### 2.3 Statistical analysis

Data analysis was conducted using SPSS software version 27.0 (31). Descriptive statistics were used to analyze participants' general characteristics and study variables. Independent *t*-test and analysis of variance (ANOVA) analyses were used to identify differences among key study variables in the first wave based on the general characteristics. Estimated means and standard errors (M ± SE) were calculated for each of the six waves, and linear trends were tested by modeling wave as a continuous variable to obtain the p level for trend.

Cyberbullying perpetration data showed substantial non-normality (skewness = 2.63, kurtosis = 9.83), whereas data for most other variables met the commonly accepted criteria of absolute skewness < 2 and kurtosis < 7 (32). Given the data non-normality, the cyberbullying perpetration analysis employed a Gamma distribution with a log link function, which is appropriate for positively skewed continuous outcomes. To examine changes in the main variables over time, we conducted repeated-measures analyses using the generalized estimating equation (GEE) approach. An unstructured working correlation matrix was specified to allow within-subject correlations across the six waves to be freely estimated without imposing a specific pattern.

Prior to the main analyses, correlation analyses (Pearson's or Spearman's, depending on variable data distributions) were conducted to examine bivariate relationships among study variables in the first wave. In addition, multicollinearity was assessed using variance inflation factor (VIF) and tolerance statistics. All VIF values were below 2.0 and all tolerance values exceeded 0.5, indicating no significant multicollinearity among the independent variables.

To examine longitudinal relationships among key variables over 6 years, GEE modeling was employed. Variables that were not significantly associated with cyberbullying perpetration in the preliminary analyses (including sex and weekend leisure and physical activity time) were excluded from the final GEE models. To examine mediation and moderated mediation effects across waves, we used the following analytic steps. Step 1-1 tested whether PSU (X) was associated with aggression (Me), Step 2 examined whether PSU was associated with cyberbullying perpetration (Y), and Step 3 evaluated mediation by testing whether both PSU and aggression were associated with Y, following a GEE-based adaptation of the Baron and Kenny (33) approach. To test moderated mediation, we incorporated the criteria of Müller et al. (34). Specifically, Step 1-2 extended Step 1-1 by adding parental structuring (Mo) and its interaction term [PSU (X) × parental structuring (Mo)] to determine whether the PSU-aggression pathway was moderated. This strategy enabled us to assess both the mediating role of aggression and the moderating influence of parental structuring within a longitudinal GEE framework.

## 3 Results

### 3.1 Baseline differences by general characteristics

Table 1 shows baseline differences in study variables according to participants' general characteristics. The 2,242 participants included 1,204 males and 1,038 females. By residential area, 930 participants lived in metropolitan areas, 977 in small/medium cities, and 335 in towns/villages. Regarding perceived SES, most participants identified themselves as having middle SES (76.5%), followed by low (13.6%) and high (9.9%) SES. In terms of primary caregiver, most participants reported their mother as the main caregiver (89.0%), followed by the father (7.5%), a grandparent (2.7%), and others (0.8%).

**Table 1:** Baseline differences in study variables according to participants' general characteristics (*n* = 2,242).

Characteristics	*n* (%)	Problematic smartphone use		Aggression		Parental structuring		Cyberbullying perpetration	
		Mean	SD	Mean	SD	Mean	SD	Mean	SD
Sex									
Male	1,204 (53.7)	2.07	0.39	1.89	0.57	3.08	0.53	0.68	1.10
Female	1,038 (46.3)	2.14	0.37	1.96	0.61	3.04	0.55	0.65	1.22
*t* (*p*)		−4.29 (< 0.001)		−2.62 (0.009^†^)		1.83 (0.067)		0.54 (0.586)	
Residential area									
Metropolitan area (a)	930 (41.5)	2.09	0.38	1.87	0.58	3.09	0.53	0.58	1.03
Small/medium city (b)	977 (43.6)	2.11	0.39	1.95	0.59	3.04	0.55	0.72	1.25
Town/village (c)	335 (14.9)	2.14	0.38	1.99	0.58	3.07	0.55	0.75	1.22
*F* (*p*)		2.85 (0.058)		7.81 (< 0.001)		2.59 (0.075)		5.01 (0.007^†^)	
				b, c>a				c>a	
Perceived SES									
Low (a)	305 (13.6)	2.16	0.39	2.08	0.57	3.01	0.56	0.67	1.13
Middle (b)	1,716 (76.5)	2.10	0.38	1.90	0.59	3.06	0.54	0.66	1.17
High (c)	221 (9.9)	2.06	0.37	1.87	0.60	3.15	0.53	0.65	1.09
*F* (*p*)		4.51 (0.011)		13.05 (< 0.001)		4.12 (0.016)		0.02 (0.985)	
		a>c		a>b, c		c>a		a>c	
Primary caregiver									
Father	169 (7.5)	2.18	0.42	2.02	0.55	2.96	0.55	0.71	0.97
Mother	1,995 (89.0)	2.10	0.38	1.91	0.59	3.08	0.54	0.67	1.18
Grandparent	60 (2.7)	2.15	0.35	1.91	0.55	2.99	0.58	0.47	0.81
Other	18 (0.8)	2.13	0.47	2.01	0.55	3.01	0.50	0.61	1.14
		2.30 (0.076)		1.88 (0.132)		2.79 (0.039)		0.68 (0.563)	

^†^Absence of equal variance assumed; SD, Standard deviation; SES, Socioeconomic status.

Cyberbullying perpetration differed significantly by residential area (*F* = 5.01, *p* = 0.007). Adolescents residing in towns/villages showed significantly higher mean frequencies of cyberbullying perpetration than those living in metropolitan areas (*p* < 0.05).

### 3.2 Longitudinal changes in study variables over 6 years

Table 2 presents longitudinal changes in study variables over the 6-year study period. All study variables—average sleep duration, weekday and weekend leisure and physical activity time, weekday and weekend smartphone usage time, peer relationships, peer aggression behaviors, PSU, aggression, parental structuring, and cyberbullying perpetration—showed significant changes over the 6-year period in the GEE analysis. Estimated M ± SE values were computed for each wave, and Wald chi-square tests revealed significant time effects for all variables (*p* < 0.001). In addition, linear trend analyses showed consistent directional patterns over time, indicating that these variables changed systematically across waves. For example, the frequency of cyberbullying perpetration decreased steadily over time (from 0.66 ± 0.02 in Wave 1 to 0.23 ± 0.02 in Wave 6), with a significant linear trend (*p* for trend < 0.001).

**Table 2:** Longitudinal changes in study variables over 6 years (*n* = 2,242).

Variables	1st wave		2nd wave		3rd wave		4th wave		5th wave		6th wave		*X* ^2^	*p*	*p* for trend
	M	SE	M	SE	M	SE	M	SE	M	SE	M	SE			
Average sleep duration (hours/day)	8.36	0.02	8.28	0.02	7.93	0.02	7.36	0.02	7.29	0.02	7.22	0.02	2,614.66	<0.001	<0.001
Weekday leisure and physical activity time	2.98	0.03	2.77	0.03	2.67	0.03	2.54	0.03	2.61	0.03	2.28	0.03	341.90	<0.001	<0.001
Weekend leisure and physical activity time	3.03	0.04	2.84	0.03	2.72	0.03	2.67	0.03	2.70	0.03	2.39	0.03	244.55	<0.001	<0.001
Weekday smartphone usage time	4.46	0.03	4.49	0.03	4.73	0.03	4.34	0.03	4.16	0.03	3.75	0.03	754.55	<0.001	<0.001
Weekend smartphone usage time	5.07	0.03	5.16	0.03	5.32	0.03	5.02	0.03	4.76	0.03	4.24	0.04	828.44	<0.001	<0.001
Peer relationships	3.13	0.01	3.11	0.01	3.11	0.01	3.12	0.01	3.07	0.01	3.02	0.01	128.35	<0.001	<0.001
Peer aggression behaviors	0.29	0.02	0.25	0.02	0.15	0.01	0.08	0.01	0.08	0.01	0.06	0.01	282.32	<0.001	<0.001
Problematic smartphone use	2.11	0.01	2.15	0.01	2.17	0.01	2.11	0.01	2.13	0.01	2.09	0.01	102.92	<0.001	<0.001
Aggression	1.92	0.01	1.90	0.01	1.86	0.01	1.85	0.01	1.89	0.01	1.80	0.01	85.15	<0.001	<0.001
Parental structuring	3.06	0.01	2.97	0.01	2.98	0.01	2.94	0.01	2.92	0.01	2.74	0.01	432.52	<0.001	<0.001
Cyberbullying perpetration	0.66	0.02	0.65	0.04	0.33	0.02	0.18	0.01	0.21	0.02	0.23	0.02	433.664	<0.001	<0.001

M, Estimated mean; SE, Standard error; *p* for trend was used to evaluate the linear change in the variable over the 6-year period.

### 3.3 Correlations among study variables

Table 3 presents correlations among study variables in the first wave of the study. PSU was significantly positively correlated with aggression (*r* = 0.409, *p* < 0.001) and cyberbullying perpetration (*r* = 0.227, *p* < 0.001). Aggression was also significantly positively correlated with cyberbullying perpetration (*r* = 0.282, *p* < 0.001), while parental structuring was significantly negatively correlated with aggression (*r* = −0.252, *p* < 0.001) and cyberbullying perpetration (*r* = −0.089, *p* < 0.001). Weekend leisure and physical activity time was the only variable that was not correlated with cyberbullying perpetration.

**Table 3:** Correlations among study variables in first wave (*n* = 2,242).

Variables	x1	x2^†^	x3^†^	x4	x5	x6	x7^†^	x8	x9	x10	y^†^
**x1**. Average sleep duration	1										
**x2**. Weekday leisure and physical activity time^†^	0.018	1									
**x3**. Weekend leisure and physical activity time^†^	0.003	0.626^***^	1								
**x4**. Weekday smartphone usage time	−0.177^***^	0.066^**^	−0.025	1							
**x5**. Weekend smartphone usage time	−0.138^***^	−0.001	−0.075^***^	0.720^***^	1						
**x6**. Peer relationships	−0.062^**^	0.101^***^	0.159^***^	−0.066^**^	−0.047^*^	1					
**x7**. Peer aggression behaviors^†^	−0.054^**^	0.084^***^	0.054^*^	0.087^**^	0.086^***^	−0.075^***^	1				
**x8**. Problematic smartphone use	−0.094^***^	−0.118^***^	−0.129^***^	0.333^***^	0.340^***^	−0.211^***^	0.134^***^	1			
**x9**. Aggression	−0.056^**^	−0.009	−0.066^**^	0.225^***^	0.205^***^	−0.376^***^	0.206^***^	0.409^***^	1		
**x10**. Parental structuring	0.090^***^	0.049^*^	0.098^***^	−0.113^***^	−0.081^***^	0.313^***^	−0.060^**^	−0.144^***^	−0.252^***^	1	
**y**. Cyberbullying perpetration^†^	−0.079^***^	0.077^***^	0.019	0.168^***^	0.164^***^	−0.063^**^	0.397^***^	0.227^***^	0.282^***^	−0.089^***^	1

Pearson's correlation coefficients are presented unless otherwise indicated;

^†^Spearman's correlation;

^*^p < 0.05,

^**^p < 0.01,

^***^p < 0.001.

### 3.4 Longitudinal mediation effect of aggression between PSU and cyberbullying perpetration

GEE was employed to identify longitudinal effects of PSU (X) and aggression (Me) on cyberbullying perpetration (Y) following the Baron and Kenny (33) framework for assessing mediation. Variables that showed significant group differences in general characteristics were dummy coded and controlled for in the model.

In Step 1-1 (PSU → Aggression), PSU showed a significant positive effect on aggression [B = 0.19, *p* < 0.001, 95% *CI* (0.18, 0.21)], indicating that higher PSU was associated with greater aggression over time. In Step 2 (PSU → Cyberbullying Perpetration), PSU was significantly and positively associated with cyberbullying perpetration [B = 0.21, *p* < 0.001, 95% *CI* (0.11, 0.32)], indicating that higher PSU was associated with greater cyberbullying perpetration over time. In Step 3 (PSU + Aggression → Cyberbullying Perpetration), aggression was significantly and positively associated with cyberbullying perpetration [B = 0.14, *p* < 0.001, 95% *CI* (0.08, 0.20)], while the association between PSU and cyberbullying perpetration remained significant [B = 0.17, *p* = 0.002, 95% *CI* (0.06, 0.27)]. These findings indicate that aggression partially mediated the relationship between PSU and cyberbullying perpetration, as evidenced by the attenuation of the PSU effect after including aggression, with the indirect effect remaining consistent across 6 years (see Table 4).

**Table 4:** Longitudinal mediation and moderated mediation models.

Variables	Step 1-1				Step 1-2				Step 2				Step 3			
	Aggression				Aggression				Cyberbullying perpetration				Cyberbullying perpetration			
	B	SE	*p*	95%*CI* (LL, UL)	B	SE	*p*	95%*CI* (LL, UL)	B	SE	*p*	95%*CI* (LL, UL)	B	SE	*p*	95%*CI* (LL, UL)
Constant	0.81	0.04	< 0.001	0.73, 0.88	1.06	0.08	< 0.001	0.90, 1.21	0.37	0.23	0.112	−0.09, 0.82	0.06	0.24	0.807	−0.42, 0.54
Residential area (small/medium city)	0.02	0.01	0.004	0.01, 0.04	0.02	0.01	0.008	0.01, 0.04	0.00	0.04	0.930	−0.07, 0.07	−0.00	0.04	0.929	−0.07, 0.07
Residential area (town/village)	0.03	0.01	0.006	0.01, 0.05	0.03	0.01	0.006	0.01, 0.05	0.02	0.05	0.679	−0.08, 0.12	0.01	0.05	0.911	−0.09, 0.10
Perceived SES (Low)	0.05	0.02	0.001	0.02, 0.08	0.05	0.02	0.002	0.02, 0.08	−0.07	0.08	0.388	−0.21, 0.08	−0.07	0.07	0.344	−0.21, 0.07
Perceived SES (Middle)	0.01	0.01	0.507	−0.02, 0.03	0.01	0.01	0.612	−0.02, 0.03	0.03	0.07	0.716	−0.11, 0.16	0.03	0.07	0.605	−0.10, 0.16
Average sleep duration	0.00	0.00	0.684	−0.00, 0.01	0.00	0.00	0.396	−0.00, 0.01	0.02	0.02	0.250	−0.02, 0.06	0.02	0.02	0.309	−0.02, 0.06
Weekday leisure and physical activity time	0.02	0.00	< 0.001	0.02, 0.02	0.02	0.00	< 0.001	0.02, 0.02	0.00	0.01	0.716	−0.02, 0.03	0.00	0.01	0.957	−0.02, 0.02
Weekday smartphone usage time	0.02	0.00	< 0.001	0.01, 0.02	0.02	0.00	< 0.001	0.01, 0.02	−0.01	0.02	0.630	−0.05, 0.03	−0.01	0.02	0.447	−0.05, 0.02
Weekend smartphone usage time	−0.01	0.00	0.005	−0.01, −0.00	−0.01	0.00	0.007	−0.01, −0.00	−0.01	0.02	0.464	−0.04, 0.02	−0.01	0.02	0.519	−0.04, 0.02
Peer relationships	−0.24	0.01	< 0.001	−0.25, −0.22	−0.22	0.01	< 0.001	−0.24, −0.21	−0.17	0.05	< 0.001	−0.26, −0.08	−0.12	0.04	0.009	−0.20, −0.03
Peer aggression behaviors	0.03	0.00	< 0.001	0.03, 0.04	0.03	0.00	< 0.001	0.03, 0.04	0.25	0.01	< 0.001	0.23, 0.27	0.24	0.01	< 0.001	0.22, 0.27
Problematic smartphone use (*X*)	0.19	0.01	< 0.001	0.18, 0.21	0.09	0.04	0.007	0.03, 0.16	0.21	0.05	< 0.001	0.11, 0.32	0.17	0.05	0.002	0.06, 0.27
Parental structuring (*M*_*o*_)					−0.10	0.03	< 0.001	−0.15, −0.05								
*X*×*M*_*o*_					0.03	0.01	0.004	0.01, 0.06								
Aggression (*M*_*e*_)													0.14	0.03	< 0.001	0.08, 0.20
QIC	979.02				977.99				977.99				859.65			

B, Unstandardized coefficient; SE, Standard error; *p, p*-value; CI, Confidence interval; LL, lower limit; UL, upper limit; QIC, Quasi-likelihood under the independence model criterion; SES, Socioeconomic status; *X*, Independent variable; *M*_*e*_, Mediator; *M*_0_, Moderator.

### 3.5 Longitudinal moderated mediation analysis: the role of parental structuring

**Figure 1:**
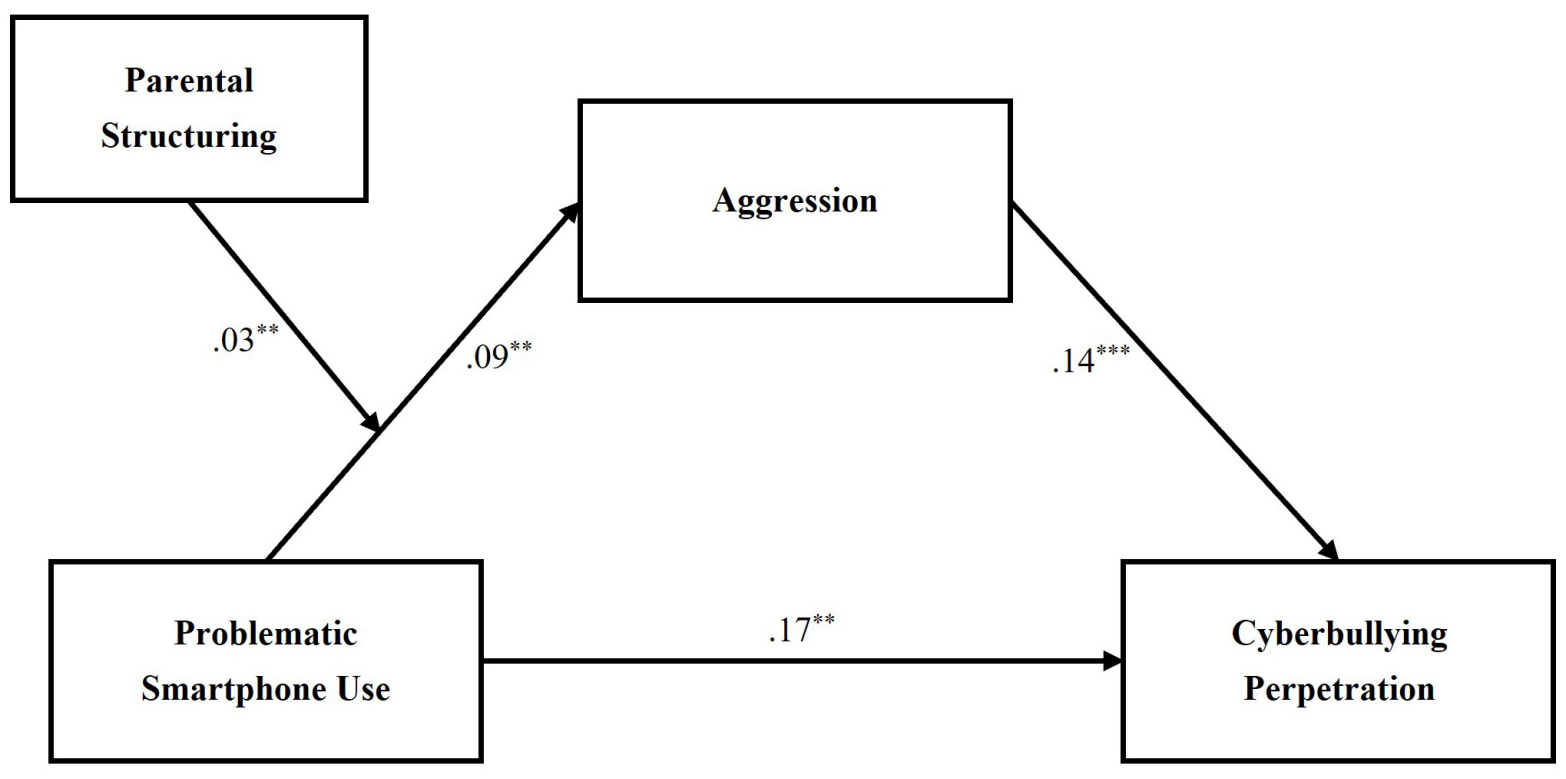
Longitudinal Moderated Mediation Model of Problematic Smartphone Use and Cyberbullying Perpetration. The association between problematic smartphone use and cyberbullying perpetration was partially mediated by aggression. Parental structuring moderated the pathway from PSU to aggression in the six-wave GEE model. All coefficients are adjusted for covariates. Unstandardized coefficients (B) are presented. **p < 0.01; ***p < 0.001.

The moderated mediation model was tested following the framework of Müller et al. (34), using GEE modeling across six waves (see Table 4). In Step 1-2, when parental structuring and the interaction term were included, PSU remained significantly associated with aggression [*B* = 0.09, *p* = 0.007, 95% *CI* (0.03, 0.16)], and parental structuring was significantly associated with aggression [*B* = −0.10, *p* < 0.001, 95% *CI* (−0.15, −0.05)]. Importantly, the interaction between PSU and parental structuring was significant [*B* = 0.03, *p* = 0.004, 95% *CI* (0.01, 0.06)], indicating that the association between PSU and aggression differed according to the level of parental structuring. In the final model (Step 3), both PSU and aggression remained significantly predictors of cyberbullying perpetration. These findings suggest that the indirect pathway from PSU to cyberbullying perpetration through aggression varied depending on the level of parental structuring in the six-wave longitudinal model (see Table 4 and Figure 1).

## 4 Discussion

This study investigated longitudinal relationships among PSU, aggression, and cyberbullying perpetration over a 6-year period using nationally representative panel data from 2,242 Korean adolescents. The findings showed that higher PSU was positively associated with greater aggression, which in turn was positively associated with higher levels of cyberbullying perpetration. Aggression partially mediated the relationship between PSU and cyberbullying perpetration, suggesting an indirect pathway. Moreover, parental structuring played a moderating role such that the association between PSU and aggression was stronger among adolescents who perceived higher levels of parental structuring.

In the present study, cyberbullying perpetration showed a consistent decline over the six-year period, with a noticeable decrease beginning around the third wave. This pattern may reflect both sociocultural and developmental factors. The study participants were aged 12–18 years and followed from early to late adolescence and the third wave coincided with the onset of the COVID-19 pandemic. The observed decline after this point is partly consistent with prior evidence indicating that cyberbullying decreased in some contexts during the pandemic, although trends varied across cultural settings (2). In addition, this pattern aligns with research showing that aggressive and peer-related problems typically peak during mid-adolescence and decline thereafter (35). These changes may reflect developmental processes during adolescence, particularly improvements in self-control and the regulation of impulsive behaviors (7, 36). In addition, this pattern may also be related to changes in the educational context, as the third wave corresponds to the transition from middle to high school in South Korea. School-related factors have been shown to influence aggression and peer-related problems among Korean adolescents (37). However, this decline may also reflect contextual or measurement-related factors, such as changes in reporting behavior, potential underreporting over time, or the possibility that individuals who continued to report cyberbullying in later waves may reflect more persistent patterns of cyberbullying involvement.

Regarding the associations among the study variables, higher levels of PSU were associated with increased aggression, which in turn was associated with increased cyberbullying perpetration, suggesting that even modest associations may have meaningful implications over time. These results are consistent with the General Aggression Model, which posits that repeated exposure to situational risk factors can strengthen aggressive cognitive, affective, and arousal responses and subsequently elevate the likelihood of aggressive behaviors, including cyberbullying perpetration (16, 17). Supporting this interpretation, studies have shown that frequent and poorly regulated smartphone use escalates online aggression and hostile communication (19, 38). Moreover, PSU use may weaken cognitive functioning that supports behavioral regulation, thereby increasing adolescents' vulnerability to hostility, anger, and aggression (13). PSU may also create a digital environment in which hostile cues are frequently encountered and normalized, reinforcing aggressive scripts and behaviors. Nuri et al. (39) have addressed that uncontrolled exposure to violent online content can influence both short- and long-term behavior, making adolescents more prone to imitate aggressive actions. These mechanisms suggest that interventions targeting PSU reduction may help to prevent escalation of aggression into cyberbullying perpetration.

Notably, our study revealed a paradoxical moderated mediation effect. Specifically, parental structuring was associated with a stronger relationship between PSU and aggression, strengthening the indirect pathway from PSU through aggression to cyberbullying perpetration. This pattern contrasts with previous findings that parental monitoring and supportive parent–child relationships are associated with lower levels of cyberbullying and aggression in children (40, 41). When interpreted through the lens of social control theory, these differing findings suggest developmental specificity. While structured parental guidance may protect younger children by enhancing their self-control, adolescents may perceive high levels of parental structuring as restricting their autonomy, thereby triggering psychological reactance that intensifies the emotional and behavioral consequences of PSU (36, 42). This interpretation may be particularly relevant in the South Korean context, where strong academic expectations often lead parents to exert heightened control over adolescents' smartphone use. Prior evidence suggests that externally imposed restrictions are less effective than self-regulated control in reducing smartphone use (43), raising the possibility that parental structuring may be experienced as coercive rather than supportive. Accordingly, the observed pattern in this study suggests that parental involvement may not uniformly function as a protective factor during adolescence and may instead be associated with greater emotional and behavioral consequences of PSU. These findings highlight the importance of developmentally tailored intervention approaches that balance guidance with adolescents' need for autonomy in addressing digital behaviors and mental health risks.

Previous research suggests that parental structuring reduces both cyberbullying perpetration and cybervictimization, but long-term follow-up shows that while it remains effective for reducing victimization, it is less effective for sustained reduction of cyberaggression (22). Moreover, our results indicate that parental structuring may not buffer against the adverse effects of PSU on adolescents. Research findings collectively suggest that the quality of parental care, rather than the level of control alone, plays a critical role during adolescence. These findings highlight potential limitations of parent-centered intervention strategies and emphasize the need for developmentally aligned approaches that respect adolescents' autonomy. Furthermore, the cyber-specific cognitions—including perceived anonymity, beliefs about the irrelevance of physical strength online, and pro-cyberbullying attitudes— have been identified as key factors driving cyberbullying perpetration (18). Recent evidence indicates that comprehensive, school-based and multicomponent programs are more effective than single-component interventions in reducing cyberbullying (44). In short, we believe that school- and community-based cyberbullying prevention programs should adopt multilevel approaches that extend beyond parent-only strategies.

This study provides a robust examination of adolescents' cyberbullying perpetration across a critical developmental period using six annual waves of nationally representative data. Also, the study results offer a nuanced, theory-based understanding of aggression mechanisms specific to cyberspace. However, a few study limitations should be noted. The findings of this study should be interpreted within the sociocultural context of South Korea, where adolescents experience high levels of digital connectivity and strong academic and parental involvement. Moreover, as this study was based on secondary data analysis, not all relevant aspects of adolescents' experiences could be fully captured. Given the well-established interconnection between victimization and perpetration (7), this omission may have influenced the observed associations within the PSU–aggression–cyberbullying perpetration pathway. In addition, several constructs were assessed using self-reports, which may introduce measurement bias. Specifically, self-reports of sensitive behaviors such as cyberbullying perpetration and problematic smartphone use may be subject to social desirability or recall bias, potentially leading to biased estimates. The parental structuring was assessed based on adolescents' perceptions, which may not fully reflect actual parental behaviors and could have influenced these associations. PSU was measured using a scale that does not capture the specific type or content of smartphone use, limiting consideration of differences in online exposure (e.g., entertainment vs. aggressive content) and other forms of online engagement. Furthermore, the inclusion criterion may limit the generalizability of the findings to adolescents with more stable longitudinal participation across waves. Finally, although the longitudinal design supports temporal inference, the use of GEE models precludes definitive causal conclusions.

As self-reported measures were used for cyberbullying behaviors and PSU, future research should incorporate more objective behavioral assessments of smartphone use patterns and online engagement (e.g., digital trace data, ecological momentary assessment, and daily logs), and it should examine additional contextual and compounding factors that may shape adolescents' online aggression trajectories. Additionally, to translate our study findings into effective cyberbullying prevention strategies, researchers should develop and evaluate developmentally tailored parenting programs that balance structure with recognition of adolescent autonomy. Such programs, along with school-based aggression management programs incorporating cyber-specific cognitive restructuring, are needed to help reduce cyberbullying perpetration among adolescents.

In conclusion. demonstrates that PSU is associated with cyberbullying perpetration indirectly through aggression, and that parental structuring is associated with a stronger linkage in this pathway during adolescence. Aggression functions as a central mechanism linking PSU to cyberbullying perpetration, consistent with prior research across diverse cultural contexts, and parental involvement alone may be insufficient to protect adolescents seeking autonomy rather than regulation. However, the pattern observed in relation to parental structuring in this study may reflect sociocultural characteristics of South Korea, where parental involvement may interact with adolescents' developmental need for autonomy. Effective cyberbullying prevention requires multilevel approaches integrating school- and community-based programs with developmentally appropriate strategies that address online behavioral risks throughout adolescence. To support the development of comprehensive intervention frameworks, future research should include objective behavioral measures and broader ecological factors to advance understanding of how cyberbullying perpetration emerges across adolescence.

## Data Availability

Publicly available datasets were analyzed in this study. This data can be found here: https://www.nypi.re.kr/archive.

## References

[1] Hinduja S Patchin JW. Bullying Beyond the Schoolyard: Preventing and Responding to Cyberbullying, 2nd Edn. Thousand Oaks, CA: Sage Publications. (2015).

[2] HuangN ZhangS MuY YuY RiemMME GuoJ. Does the COVID-19 pandemic increase or decrease the global cyberbullying behaviors? a systematic review and meta-analysis. Trauma Violence Abuse. (2024) 25:1018–35. doi: 10.1177/1524838023117118537177992 PMC10185480

[3] Korea Communications Commission and National Information Society Agency. 2023 Survey on Cyberbullying in Korea (in Korean) (2024). Available online at: https://www.kmcc.go.kr/user.do?mode=viewandpage=A02060400anddc=K02060400andboardId=1030andcp=1andboardSeq=60296 (Accessed February 12, 2026).

[4] BarlettCP BennardiC WilliamsS ZlupkoT. Theoretically predicting cyberbullying perpetration in youth with the BGCM: unique challenges and promising research opportunities. Front Psychol. (2021) 12:708277. doi: 10.3389/fpsyg.2021.70827734659022 PMC8513570

[5] LiJ HeskethT. Experiences and perspectives of traditional bullying and cyberbullying among adolescents in Mainland China—implications for policy. Front Psychol. (2021) 12:672223. doi: 10.3389/fpsyg.2021.67222334295284 PMC8290073

[6] Agustiningsih N Yusuf A Ahsan A. Relationships among self-esteem, bullying, and cyberbullying in adolescents: a systematic review. J Psychosoc Nurs Ment Health Serv. (2024) 62:11–7. doi: 10.3928/02793695-20231013-01 37879085

[7] Marciano L Schulz PJ Camerini A-L. Cyberbullying perpetration and victimization in youth: a meta-analysis of longitudinal studies. J. Comput Mediat Commun. (2020) 25:163–81. doi: 10.1093/jcmc/zmz031

[8] Quintana-Orts C Rey L Neto F. Beyond cyberbullying: investigating when and how cybervictimization predicts suicidal ideation. J Interpers Violence. (2022) 37:935–57. doi: 10.1177/0886260520913640 32345110

[9] KimD LeeY LeeJ NamJK ChungY. Development of Korean smartphone addiction proneness scale for youth. PLoS ONE. (2014) 9:e97920. doi: 10.1371/journal.pone.009792024848006 PMC4029762

[10] National Information Society Agency. 2024 Smartphone Overdependence Survey [in Korean] (2024). Available online at: https://www.nia.or.kr/site/nia_kor/ex/bbs/View.do?bcIdx=27831andcbIdx=65914andparentSeq=27831 (Accessed February 12, 2026).

[11] Olson JA Sandra DA Colucci ÉS Bikaii AA Chmoulevitch D Nahas J . Smartphone addiction is increasing across the world: a meta-analysis of 24 countries. Comput Hum Behav. (2022) 129:107138. doi: 10.1016/j.chb.2021.107138

[12] Chun J Lee HK Jeon H Kim J Lee S. Impact of COVID-19 on adolescents' smartphone addiction in South Korea. Soc Work Public Health. (2023) 38:268–80. doi: 10.1080/19371918.2022.2134252 36227775

[13] Fekih-RomdhaneF MalaebD Sarray El DineA ObeidS HallitS. The relationship between smartphone addiction and aggression among Lebanese adolescents: the indirect effect of cognitive function. BMC Pediatr. (2022) 22:735. doi: 10.1186/s12887-022-03808-y36572845 PMC9791769

[14] Hussain Z Kircaburun K Savci M Griffiths MD. The role of aggression in the association of cyberbullying victimization with cyberbullying perpetration and problematic social media use among adolescents. J Concurrent Disord. (2023) 10:1–10. doi: 10.54127/AOJW5819

[15] Ban J Kim D. Structural relationship among aggression, depression, smartphone dependency, and cyberbullying perpetration. Child Youth Serv Rev. (2024) 166:107976. doi: 10.1016/j.childyouth.2024.107976

[16] Anderson CA Bushman BJ. Human aggression. Annu Rev Psychol. (2002) 53:27–51. doi: 10.1146/annurev.psych.53.100901.135231 11752478

[17] Barlett CP Kowalski RM Wilson AN. Meta-analyses of the predictors and outcomes of cyberbullying perpetration and victimization while controlling for traditional bullying perpetration and victimization. Aggress Violent Behav. (2023) 61:101886. doi: 10.1016/j.avb.2023.101886

[18] NagataJM YangJH SinghG KissO GansonKT TestaA . Cyberbullying and sleep disturbance among early adolescents in the U.S. Acad Pediatr. (2023) 23:1220–5. doi: 10.1016/j.acap.2022.12.00736581100 PMC10291005

[19] Wang Z Jiang S. Influence of parental neglect on cyberbullying perpetration: moderated mediation model of smartphone addiction and self-regulation. Health Soc Care Commun. (2022) 30:2372–82. doi: 10.1111/hsc.13787 35298055

[20] Skinner E Johnson S Snyder T. Six dimensions of parenting: a motivational model. Parenting Sci Pract. (2005) 5:175–235. doi: 10.1207/s15327922par0502_3

[21] ZhangY ChenC TengZ GuoC. Parenting style and cyber-aggression in Chinese youth: the role of moral disengagement and moral identity. Front Psychol. (2021) 12:621878. doi: 10.3389/fpsyg.2021.62187833679537 PMC7933004

[22] Wang L Jiang S. Effectiveness of parent-related interventions on cyberbullying among adolescents: a systematic review and meta-analysis. Trauma Violence Abuse. (2023) 24:3678–96. doi: 10.1177/15248380221137065 36458864

[23] Kowalski RM Giumetti GW Schroeder AN Lattanner MR. Bullying in the digital age: a critical review and meta-analysis of cyberbullying research among youth. Psychol Bull. (2014) 140:1073. doi: 10.1037/a0035618 24512111

[24] Viner RM Gireesh A Stiglic N Hudson LD Goddings AL Ward JL . Roles of cyberbullying, sleep, and physical activity in mediating the effects of social media use on mental health and wellbeing among young people in England: a secondary analysis of longitudinal data. Lancet Child Adolesc Health. (2019) 3:685–96. doi: 10.1016/S2352-4642(19)30186-5 31420213

[25] National Youth Policy Institute. Korean Children and Youth Panel Survey 2018 (KCYPS 2018) [Data set] (2024). Available online at: https://www.nypi.re.kr/archivehttps://www.nypi.re.kr/archive (Accessed February 12, 2026).

[26] Cho BH Lim KH. Development and validation of emotional or behavioral problems scale. Korean J Couns Psychother. (2003) 15:729–46.

[27] Kim T Lee E. Validation of the Korean version of parents as social context questionnaire for adolescents: PSCQ_KA. Korean J Youth Stud. (2017) 24:313–33. doi: 10.21509/KJYS.2017.03.24.3.313

[28] Lee S Kang J Lee W. A Study on the Types and Countermeasures of Youth Cyber Violence. Seoul: Korean Institute of Criminology (2015).

[29] Bae SM Hong JY Hyun MH. A validation study of the peer relationship quality scale for adolescents. Korean J Youth Stud. (2015) 22:325–44.

[30] Kim JK Baek HJ Lim HJ Lee GO. Korean Children and Youth Panel Survey 2010 I: Project Report (2010). Available online at: https://repo.kicce.re.kr/handle/2019.oak/833 (Accessed February 12, 2026).

[31] Corp IBM. IBM SPSS Statistics for Windows, Version 27.0. Armonk, NY: IBM Corp (2020).

[32] Kline RB. Principles and Practice of Structural Equation Modeling, 4th Edn. New York, NY: Guilford Press. (2016).

[33] Baron RM Kenny DA. The moderator–mediator variable distinction in social psychological research: conceptual, strategic, and statistical considerations. J Pers Soc Psychol. (1986) 51:1173–82. doi: 10.1037//0022-3514.51.6.1173 3806354

[34] Muller D Judd CM Yzerbyt VY. When moderation is mediated and mediation is moderated. J Pers Soc Psychol. (2005) 89:852–63. doi: 10.1037/0022-3514.89.6.852 16393020

[35] Cheong J Chang EH. A study on offending and persistence in juvenile delinquency: comparing the effects of family, school, and propensity factors. Korean Criminol Rev. (2023) 34:163–92. doi: 10.36889/KCR.2023.9.30.3.163

[36] Hirschi T. Causes of Delinquency, 2nd Edn. London: Routledge (2017).

[37] Song J. Patterns and explanations of delinquency among Korean youth using general strain theory. Child Youth Serv Rev. (2020) 114:105080. doi: 10.1016/j.childyouth.2020.105080

[38] Abaido GM. Cyberbullying on social media platforms among university students in the United Arab Emirates. Int J Adolesc Youth. (2020) 25:407–20. doi: 10.1080/02673843.2019.1669059

[39] Nuri C Direktör C Arnavut A. The mediation effects of smartphone addiction on the relationship between aggression and nomophobia. World J Educ Technol Curr Issues. (2021) 13:106–14. doi: 10.18844/wjet.v13i1.5403

[40] Özdemir Y Vazsonyi AT Çok F. Parenting processes, self-esteem and aggression: a mediation model. Eur J Dev Psychol. (2017) 14:509–32. doi: 10.1080/17405629.2016.1240674

[41] Baldry AC Sorrentino A Farrington DP. Cyberbullying and cybervictimization versus parental supervision, monitoring and control of adolescents' online activities. Child Youth Serv Rev. (2019) 96:302–7. doi: 10.1016/j.childyouth.2018.11.058

[42] YunJ HanG SonH. Protective and risk factors of problematic smartphone use in preteens using panel study on Korean children. Front Psychiatry. (2022) 13:981357. doi: 10.3389/fpsyt.2022.98135736061301 PMC9437291

[43] Hur YJ. The effect of parents' smartphone usage time limit method on the reduction of smartphone usage time in upper elementary school students. J Knowl Inf Technol Syst. (2023) 18:1487–96.

[44] Gaffney H Farrington DP Ttofi MM. Examining the effectiveness of school-bullying intervention programs globally: a meta-analysis. Int J Bullying Prev. (2019) 1:14–31. doi: 10.1007/s42380-019-0007-4

